# The Role of IL-7 and IL-7R in Cancer Pathophysiology and Immunotherapy

**DOI:** 10.3390/ijms231810412

**Published:** 2022-09-08

**Authors:** Chunli Wang, Lingzu Kong, Seokmin Kim, Sunyoung Lee, Sechan Oh, Seona Jo, Inhwan Jang, Tae-Don Kim

**Affiliations:** 1 Immunotherapy Research Center, Korea Research Institute of Bioscience and Biotechnology, Daejeon 34141, Korea; 2 Department of Biochemistry, College of Natural Sciences, Chungnam National University, Daejeon 34134, Korea; 3 Department of Functional Genomics, KRIBB School of Bioscience, Korea University of Science and Technology, Daejeon 34113, Korea; 4 Department of Life Sciences, Korea University, Seoul 02841, Korea

**Keywords:** IL-7, IL-7R signal, cancer, immunotherapy

## Abstract

Interleukin-7 (IL-7) is a multipotent cytokine that maintains the homeostasis of the immune system. IL-7 plays a vital role in T-cell development, proliferation, and differentiation, as well as in B cell maturation through the activation of the IL-7 receptor (IL-7R). IL-7 is closely associated with tumor development and has been used in cancer clinical research and therapy. In this review, we first summarize the roles of IL-7 and IL-7Rα and their downstream signaling pathways in immunity and cancer. Furthermore, we summarize and discuss the recent advances in the use of IL-7 and IL-7Rα as cancer immunotherapy tools and highlight their potential for therapeutic applications. This review will help in the development of cancer immunotherapy regimens based on IL-7 and IL-7Rα, and will also advance their exploitation as more effective and safe immunotherapy tools.

## 1. Introduction

Interleukin 7 (IL-7) is a cytokine necessary for the adaptive immune system, which is critical for B cell development [1,2,3] as well as proliferation and survival of memory and naive T cells, and T cell development in the thymus [4]. IL-7 performs its biological functions mainly through the activation of the IL-7 receptor (IL-7R) [5]. IL-7R is a heterodimer that is composed of the IL-7R α chain (CD127) and the common γ chain (CD132, IL-2Rγ) shared by multiple cytokines such as IL-2, IL-7, IL-4, IL-9, IL-15, and IL-21 [1]. IL-7 and IL-7Rα promote cell survival and inhibit cell apoptosis mainly by the activation of Janus kinase (JAK), signal transduction factor and transcription activator 5 (STAT5), and the phosphatidylinositol 3-kinase (PI3K)—protein kinase B (AKT)-mediated signal pathway [6,7,8]. IL-7 has strong immunomodulatory effects, which can directly or indirectly act on tumor cells and exert anti-tumor effects by enhancing tumor eradication or adoptive immunity [9]. Conversely, IL-7 also has potential pro-tumor effects via the activation of downstream JAK/STAT5 and PI3K–AKT pathways [10,11,12]. IL-7 is closely associated with tumor development and has been used in clinical research and treatment [5,13]. Therefore, it is necessary to provide a comprehensive review of all the available information for a better comprehension of the biological functions of IL-7 and IL-7Rα in immunity and cancer progression, and their potential applications in cancer immunotherapy, which may be useful for their utilization as more effective and safe immunotherapy tools. In this review, we summarize the roles of IL-7, IL-7Rα, and their downstream signaling pathways in immunity and cancer development. We then summarize the recent progress in the use of IL-7 and IL-7Rα in cancer immunotherapy and discuss their potential for therapeutic applications.

## 2. Biology and Functions of IL-7 and IL-7R

### 2.1. Biology and Functions of IL-7

IL-7 is widely expressed in many tissues, including lymphoid organs such as the bone marrow, thymus, lymph nodes, and spleen, as well as in non-lymphoid sites such as the skin, lung, intestine, and liver [14,15]. However, IL-7 is predominantly secreted by the bone marrow, thymus, and lymph nodes to maintain the body’s immune self-stability [5]. The human IL-7 gene, located on chromosome 8, has 534 bp, contains 6 exons and 5 introns, and encodes a protein of 177 amino acids with a molecular weight of approximately 20 kDa. The active form of IL-7 encodes a 25 kDa, single-chain glycoprotein that is predicted to form a structure containing four α-helices with a hydrophobic core [2].

IL-7 can promote early B cell proliferation in mice, in vitro, and can also promote the growth of precursor B cells [16,17]. IL-7 knockout mice showed developmental retardation of bone marrow, inability to convert from pro-B cells to pre-B cells, lack of mature T cells and B cells, and a 20-fold reduction in thymic cells, indicating that IL-7 plays a crucial role in the development and maturation of the bone marrow, and the central T and B cells of the thymus [18]. Moreover, IL-7 could increase the viability of naive T cells in the absence of antigenic stimulation, suggesting that it plays an essential role in protecting the naive T cell repertoire [19,20,21]. It also plays a vital role in increasing memory T cell survival and expansion [22,23]. The deficiency of IL-7 and its receptor affects the development of B cells, T cells, natural killer (NK) cells, monocytes, macrophages, dendritic cells, and innate lymphoid cells, indicating that IL-7 plays crucial regulatory roles in the entire immune system [24].

### 2.2. Biology and Functions of IL-7Rα

**Figure 1:**
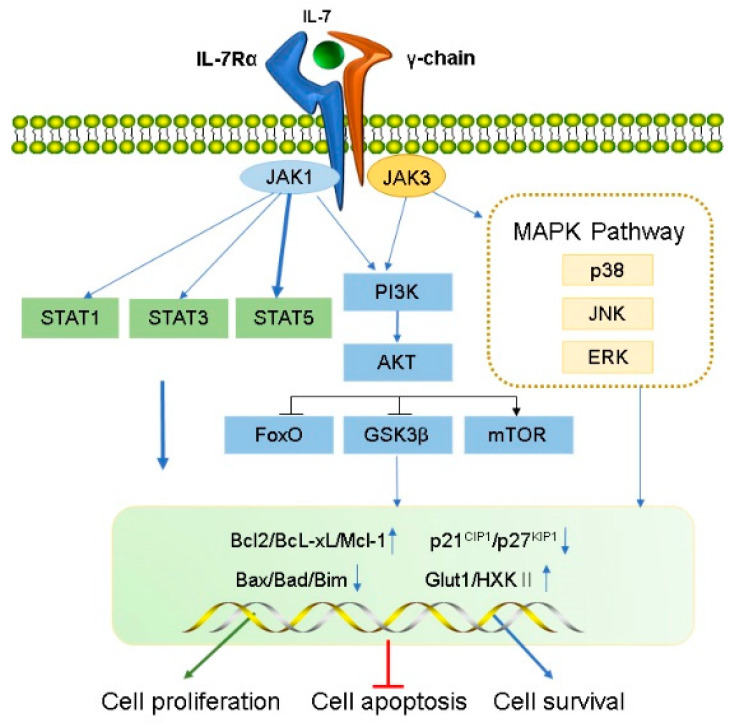
Transduction of IL-7 signaling pathway. IL-7 induces the activation of IL-7R downstream signaling pathway kinases, including JAK1 (linked to IL-7Rα) and JAK3 (linked to common γC), STAT1, STAT3, STAT5, PI3K, AKT, and MAPK. IL-7 signal transduction promotes cell proliferation and survival and inhibits apoptosis by regulating gene expression levels in the nucleus, including a decrease in pro-apoptotic factors (such as Bad and Bax) and cell cycle inhibitors (p21^CIP1^ and p27^KIP1^) and an increase in anti-apoptotic factors (such as Bcl-XL, Bcl-2, and McL-1) and glucose metabolism regulators (Glut1, HXKⅡ).

IL-7 function is mediated by the IL-7R, a heterodimer consisting of the IL-7R α chain (CD127) and a common γ chain (CD132, IL-2Rγ). The human IL-7Rα gene, located on chromosome 5, contains 1380 bp, includes 8 exons and 7 introns, and encodes for a protein of 459 amino acids with a molecular weight of approximately 49.5 kDa [2]. IL-7Rα is expressed in hematopoietic cells, particularly the lymphoid lineage, including fetal NK/dendritic precursors, mature T cells and bone marrow macrophages, and developing T cells and B cells. Human marrow stromal cells [25], endothelial cells [26], normal human intestinal epithelial cells, and several malignant tumor cell lines containing breast cancer, melanoma, leukemia, lung cancer and cutaneous T cell lymphoma [27,28,29,30] were all found to express IL-7Rα. IL-7Rα has two forms, membrane-bound IL-7Rα and soluble IL-7Rα (sIL-7Rα), with different biological functions [31,32]. sIL-7Rα competes with membrane IL-7R to reduce excessive IL-7 consumption and antagonizes IL-7 signaling, hence enhancing the biological activity of IL-7 when cytokines are restricted [33]. In addition, sIL-7R directly bind to IL-2Rγ on membrane surface and inhibit IL-7 signaling in IL-2Rγ-positive cells [34]. Previous studies found that sIL-7Rα aggravate autoimmune diseases [33,35,36]. However, sIL-7Rα concentrations were demonstrated to be significantly enhanced in the serum of HIV-positive patients, and high concentrations of sIL-7Rα inhibit IL-7-mediated CD8+ T cell proliferation, indicating that sIL-7Rα may play dual regulatory roles in vivo [33,37,38]. Membrane-bound IL-7Rα promotes cell growth and proliferation, and it inhibits apoptosis by regulating the IL-7 signaling pathway [37]. During this process, IL-7 first binds to IL-7Rα and then recruits IL-2Rγ to form a ternary signaling complex [39], which activates two main downstream signaling pathways, the JAK/STAT5 and the PI3K–AKT signal pathways [6,7,8]. Furthermore, IL-7 also induces the activation of mitogen-activated protein kinases (MAPK) pathway [40] (Figure 1).

#### 2.2.1. JAK/STAT5 Signaling Pathway

IL-7 binds to IL-7Rα, triggering activation of the IL-7Rα-associated tyrosine kinase, JAK 1 (linked to IL-7Rα), and JAK3 (linked to common γC). The activated JAK protein phosphorylates a specific motif on the IL-7Rα chain to form a binding site for STAT5 (a signaling molecule containing Src homologous 2 (SH2) domains), and then binds and phosphorylates STAT5, which forms a dimer and enters the nucleus.

During this process, a series of genes that modulate cell growth and survival in the nucleus is affected, as well as other pathways such as PI3K–AKT and MEK/extracellular signal-regulated kinase (ERK) are activated. For example, anti-apoptotic proteins Bcl-XL, Bcl-2, McL-1 belonging to the bcl-2 protein family are up-regulated, and pro-apoptotic proteins (BAX, BAD) are down-regulated, which improve the survival of T cells in vivo [41]. IL-7 signaling can maintain survival of memory CD8 T cells by mediating STAT5 and STAT3 activation [42]. However, overexpression of Bcl-2 and Bcl-XL did not prevent effector cell death during lymphocytic choriomeningitis virus infection [43], suggesting that activation of other signaling pathways downstream of IL-7R are crucial for maintaining the survival of memory cell precursors. Correspondingly, basal levels of IL-7 can also regulate the number of memory CD8 T cells formed [44]. Furthermore, IL-7 mediates activation of STAT5 and is necessary for T cell proliferation [45], differentiation [46] and survival [47,48]. It also regulates T cell cytotoxicity [47] and drug resistance [49,50]. Additionally, IL-7 not only leads to IL-7-dependent activation of STAT1 and STAT5 in the presence of lymphopenia, but also enhances T cell response to type-I IFN by regulating STAT1 protein expression level [51,52]. In addition, STAT1 overexpression was related to reduce survival in CD4+ T cells undergoing lymphocytopenia-induced proliferation [52]. These results suggest that STAT1 is involved in the process by which IL-7 regulates T cell survival. IL-7 also activates STAT1 and STAT3 which promote B cell precursor acute lymphoblastic leukemia proliferation [53] and survival of B cell progenitors [54], respectively. Furthermore, The JAK/STAT pathway not only activates the family of cytokine signaling inhibitor proteins (SOCS) but can also be inhibited by them to form a negative feedback loop [55,56]. SOCS proteins inhibit cytokine signaling either by competing with STAT5 to inhibit JAK [57] or by proteasomal degradation of targeted signaling proteins [58,59,60]. 

#### 2.2.2. PI3K/AKT/mTOR Signaling Pathway

Activated IL-7Rα stimulates JAK1/3, and then phosphorylates the P85 subunit of PI3K to activate PI3K and produces the second messenger phosphatidylinositol-(3,4,5)-trisphosphate (PIP3) on the plasma membrane. PIP3 binds to the signaling proteins AKT and (3-phosphoinositide-dependent protein kinase 1) PDK1 (containing Pleckstrin homology domain) and then promotes PDK1 to phosphorylate Ser308 of the AKT protein, thereby activating AKT. IL-7/IL-7R pathways mediate the main downstream targets of AKT such as glycogen synthase kinase (GSK, inhibited), forkhead box O (FoxO, inhibited), and mammalian target of rapamycin (mTOR, activated) [61]. AKT phosphorylates tuberous sclerosis complex 1/2 and prevents negative regulation of small GTP-binding proteins Rheb, resulting in enrichment of Rheb and activation of the mTOR complex (mTORC1) which promotes cell survival and proliferation by inhibiting Bad, Bim, Bax, p21^CIP1^ and p27^KIP1^ and activating Cdk2 [62,63]. Additionally, IL-7 increases the expression of glucose transporter 1 (Glut1) and glycolytic enzyme hexokinase II (HXKII), thereby increasing glucose uptake [64,65] and regulating glucose utilization depending on the PI3K/AKT signaling pathway [66]. IL-7 mediates the proliferation and activation of T cells in mice and is attenuated by PI3K inhibitors [67]. Furthermore, PI3K/AKT pathway is inhibited by PTEN; inhibitors of this pathway are critical for pro-B cell development [68]. IL-7 may promote adipose-derived stem cell differentiation by increasing AKT phosphorylation [69]. Therefore, the PI3K/AKT pathway is essential for powerful IL-7 signal transduction in the cell cycle.

#### 2.2.3. MAPK Pathway

Early studies have shown that IL-7 activates MAPK, containing p38 kinase, c-Jun N-terminal kinase (JNK), and ERK [40]. IL-7-induced cell proliferation could be mediated by the inhibition of the downstream effector MAPK-activated proteinase 2, further verifying that IL-7 activates this pathway [70]. Specific P38 inhibitors inhibit IL-7-induced T cell proliferation, suggesting that the P38 MAPK pathway plays a vital role in IL-7 signal transduction [70]. Additionally, IL-7 withdrawal blocks the activation of P38 and JNK kinases, leading to IL-7-dependent thymocyte death [71]. IL-7 rescues rapamycin-induced apoptosis of B-cell precursor acute lymphoblastic leukemia-acute lymphoblastic leukemia (ALL) cells by upregulating MEK/ERK [72]. Hence, the MAPK pathway may play a vital role in regulating cell development via IL-7-mediated signal transduction.

## 3. Effects of IL-7 and IL-7Rα in Cancer

### 3.1. Anti-Tumor Effects of IL-7 and IL-7Rα

**Figure 2:**
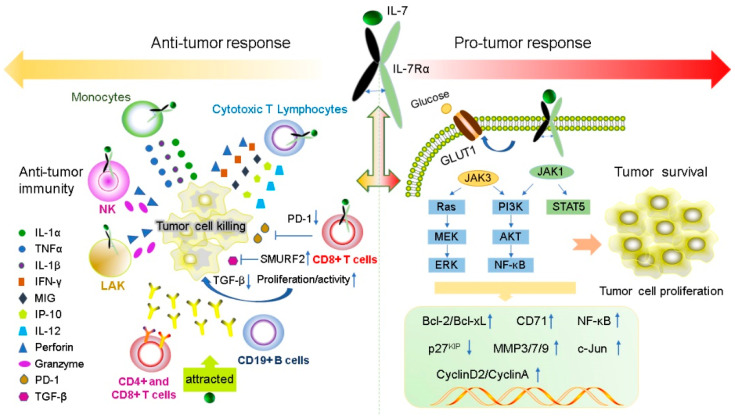
IL-7 and IL-7R have both pro- and anti-tumor functions. IL-7 plays anti-tumor roles by regulating immune cells to release cytokines such as IFN-γ, IL-1β, IL-1α and TNF-α. In contrast, IL-7 can promote the proliferation and survival of tumor cells by binding to the IL-7R to active JAK/STAT5, the PI3K/AKT and Ras/ERK signaling pathways to regulate gene expression levels of Bcl-2, Bcl-XL, CyclinA, CyclinD2, and p27^kip^.

IL-7 has a powerful immunomodulatory effect, which can directly or indirectly act on tumor cells and exert anti-tumor effects by enhancing tumor eradication or adaptive immunity. The expression levels of IL-7 and IL-7Rα are important for normal T cell development and sustaining the homeostasis of the immune system [18,73,74]. IL-7 enhances the cytotoxicity of NK, NKT, lymphokine-activated killer (LAK) cells, monocytes, and cancer-specific cytotoxic T lymphocytes (CTLs). It induces CTL to secrete perforin in a STAT5-dependent manner [47] and stimulates the expression of interferon-gamma (IFN-γ), mitogen-inducible gene (MIG), IL-12, and IFN-γ-induced protein 10 (IP-10) [75,76]. IL-7 can also increase the cytolytic functions of NK cells [77] and CTL [78] by increasing FasL mRNA and protein expression in the membrane. Furthermore, IL-7 increases the amount of CD4+, CD8+ T, cells and CD19+ B cells to promote antibody-dependent cell-mediated cytotoxicity; moreover, it also enhances the response of antigen-specific CD8+ T cells [79] and improve the recovery of CD4+ T cells after chemotherapy in solid tumors [80]. IL-7 inhibits melanoma growth by promoting the secretion of the cytokines IL-1β, IL-1α, and tumor necrosis factor-α (TNF-α) from monocytes [81]. IL-7 enhances the antitumor effect of IFN-γ in rat gliomas [9]. IL-7 restores the activity of CD8+ T cells by decreasing the expression of exhaustion marker PD- 1 [82,83]. Some tumors secrete TGF-β, which inhibits the proliferation of CD8+ T cells via SMAD proteins. IL-7 can reverse this inhibition by inducing the expression of SMAD ubiquitination regulatory factor 2 (SMURRF2) [82,83] (Figure 2).

Recent studies suggest that IL-7Rα may be a beneficial prognostic marker for patients with lung adenocarcinoma (LUAD). Survival analysis showed that IL-7Rα expression is an independent prognostic factor for LUAD. IL-7Rα expression is positively correlated to the overall and progression-free survival in patients with LUAD, and negatively correlated to tumor size. IL-7Rα inhibits the growth of tumor cells by affecting the percentage of infiltrating cells in the tumor immune microenvironment. Thus, IL-7Rα may also be a possible therapeutic target for LUAD [84].

### 3.2. Pro-Tumor Effects of IL-7 and IL-7Rα

The expression levels of IL-7 and IL-7Rα are important for normal T cell development and preservation of homeostasis in the immune system [18,73,74]. IL-7 and IL-7Rα have bidirectional regulatory effects on tumors. IL-7 transgenic mice induced T cell dysplasia, characterized by decreased CD4+ CD8+ (double-positive) thymocytes and lymphoproliferative diseases such as B and T cell lymphoma [85]. Xenotransplant models of human T-ALL have shown that IL-7 promotes the formation of human T-ALL, providing a new method for the treatment of T-ALL by targeting IL-7/IL-7R signal transduction [86]. Moreover, during normal T-cell development, IL-7 exerts as an anti-apoptotic factor by upregulating the of Bcl-2 expression [24]. A similar situation appears in T-ALL cells, IL-7 not only upregulates the expression of Bcl-2 and down-regulates the cyclin-dependent kinase inhibitor p27^kip1^ in T-ALL cells to avoid apoptosis, but also leads to continuing reaction of cyclin D2 and cyclin A during cell cycle progression [87,88]. All gamma-cytokines promote the proliferation of primary T-ALL cells, and IL-7 is the most potent cytokine that induces the proliferation of leukemia cells [89]. IL-7 mainly affects the proliferation and apoptosis of T-ALL cells by activating the JAK/STAT5 and PI3K/Akt/mTOR signaling pathways, leading to upregulation of transferrin receptor CD71, glucose transporter Glut1, glucose uptake and mitochondrial integrity [50,55,66,90,91]. In addition to its role in T-ALL formation, IL-7 also affects the invasion and growth of other tumor cells. For instance, expression of IL-7 is closely correlated with poor prognosis in prostate cancer (PCa) [92]. The IL-7/IL-7R pathway promotes the invasion and migration of PCa cells by activating the AKT/NF-κB pathway and regulating the expression of metalloproteinases (MMP 3 and 7), and it promotes the invasion and migration of bladder cancer cells via NF-KB-mediated upregulation of MMP-9 expression [12,93]. Furthermore, IL-7 can also induce the upregulation of cyclin D1 by modulating the c-FOS/c-Jun pathway, thereby promoting the proliferation of lung cancer cells [94]. Therefore, these studies also demonstrated the potential of IL-7 to promote cancer development.

Previous studies have shown that gain-of-function in IL-7R plays a key role in the generation of human T-ALL [95] and specific mutations in IL-7R specifically enhances steroid-resistance in T-ALL. Steroid resistance occurs due to mutations in IL-7R or other signaling molecules in this pathway which activate downstream MEK-ERK and AKT components, thereby upregulating the expression of MC1 and Bcl-XL, leading to a strong anti-apoptotic response. In addition, MEK-ERK and AKT signaling pathways also inhibit BIM, which is an important steroid-induced cell death molecule, and GSK3B, which is an important regulator of pro-apoptotic BIM. However, IL-7R signaling inhibitors can restore steroid resistance [96]. In addition, the abnormal expression of wild-type IL-7R can lead to the occurrence of disease and even carcinogenesis. Insufficient IL-7R expression due to IL-7R gene polymorphism results in decreased T cells numbers; however, B cells numbers remain unaffected [97]. Overexpression of IL-7R also leads to potential thymocyte self-renewal and thymic hyperplasia related to proliferation of T cell precursors, which in turn infiltrates the lymph nodes, spleen, and bone marrow, ultimately resulting in fatal leukemia in a dose-dependent manner [98] (Figure 2).

## 4. Application of IL-7 and IL-7R in Cancer Immunotherapy

**Figure 3:**
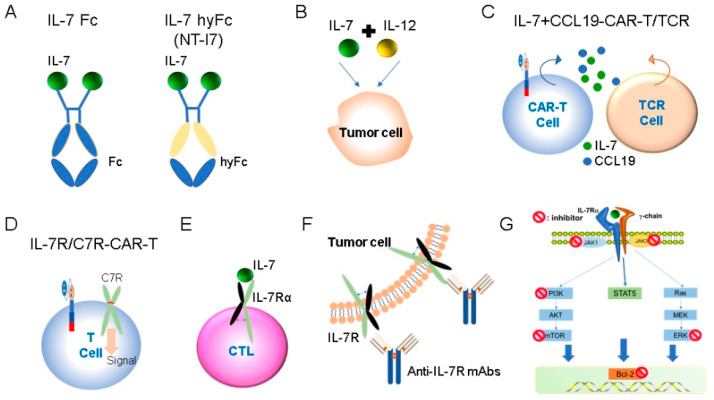
Application of IL-7 and IL-7R in cancer immunotherapy. (**A**) Recombinant IL-7 administration. (**B**) Combination use of IL-7. (**C**) CAR-T and TCR T cell expressed IL-7. (**D**) CAR-T expressing engineered IL-7R or C7R. (**E**) CTL expressed engineered IL-7R. (**F**) Anti-IL-7R monoclonal antibodies. (**G**) IL-7R pathway signaling inhibitors.

IL-7 and IL-7R are thought to be critical for regulating the impaired immune system of cancer patients. Cytokine-based immunotherapy has been used in cancer treatment for many years. IL-2 and IL-15 are effective in supporting cancer immunotherapy [99,100], but they also have some side effects. IL-2 induces severe biochemical abnormalities in the kidney and liver [100,101,102]. IL-15 is less toxic than IL-2 but can cause fever and a drop in blood pressure. In previous reports of clinical trials, low toxicity was observed, including transient fever and systemic lupus erythematosus in patients injected subcutaneously with different doses of IL-7. Meanwhile, previous research has found that the use of IL-7 accelerated immune reestablishment in mouse models [103] and that IL-7 was well tolerated in early clinical trials [104,105]. Therefore, IL-7 might mitigate the side effects of using IL-2 in cancer immunotherapy. IL-7R-mediated signaling pathways have been widely used in fluid tumors and some solid tumor models (Figure 3).

### 4.1. Recombinant IL-7 Administration in Cancer Immunotherapy

#### 4.1.1. Recombinant IL-7 Administration

IL-7 exerts a crucial role in adoptive T cell therapy. T cell receptor (TCR)-modified T cells are usually activated by CD3 stimulation, but this is not the optimal treatment. TIL1383I TCR-modified T cells (gene-edited T cells expressing TCR reactive to melanoma antigen, tyrosinase) were co-cultured with IL-7 in the absence of CD34 activation. It was found that TIL1383I TCR-modified T cells were enriched with naive and memory stem cells. The experiments have proved that IL-7 and TIL1383I TCR-modified T cells without CD3-stimulated co-culture responded well to melanoma, and they overcame the disadvantages of conventional CD3-activated T cells, providing feasible strategies for improving survival in melanoma and other malignant tumors [106].

Since cytokines have a short half-life in the body and can act in an autocrine or paracrine manner over short distances, the fusion of the IgG Fc domain with IL-7 mitigated this problem. Recombinant IL-7-Fc (Chimerigen) has also been used in adoptive cell therapy against melanoma. In a lymphocytic mouse melanoma model, IL-7-Fc therapy increased inhibition of tumor growth [107]. Another type of recombinant IL-7 (NT-I7, NeoImmuneTech/Genexine) significantly enhanced the antitumor activity of T cells and was more stable and efficient than recombinant hIL-7 [108]. NT-17 is currently used in oncology, immunology and infectious diseases.

Treatment-related lymphocytopenia is an important problem in cancer patients. NT-I7 increases T cell counts in lymphoid organs and enhances survival in mouse models following treatment with radiotherapy (RT) and RT+ temozolomide. NT-I7 therapy improves the immune response by enhancing the percentage of cytotoxic CD8+ T cells to regulatory T (Treg) cells within the tumor microenvironment (TME). Treg cells have immunosuppressive effects in brain TME [109], and the possible reasons for the failure of brain tumor immunotherapy include the survival advantage of Tregs [110]. IL-7 treatment breaks the survival advantage of Tregs, but IL-2 treatment does not have this ability. IL-2 can promote Tregs proliferation in vitro [111], and peripheral homeostasis of Treg cells in vivo is more dependent on IL-2 than on other γc cytokines [112]. Correspondingly, IL-2 treatment during immune reconstitution after chemotherapy significantly increases the number of Tregs [113]. In short, this evidence suggests that IL-7 is more helpful than IL-2 in improving current inadequacies in brain tumor therapy. In addition, a phase I/II trial (NCT03687957) evaluating the efficacy of NT-I7 in patients with superior glioma is currently underway [110]. The strategies mentioned above are shown in Figure 3A. Based on its promising therapeutic effects, IL-7 and recombinant IL-7 are being used in various clinical trials for cancer immunotherapy (Table 1).

#### 4.1.2. Combination Use of IL-7

As of 2021, the only immunotherapy approved by the Food and Drug Administration (FDA) for metastatic castration-resistant prostate cancer is sip-T. IL-7 can be used in combination with sip-T to provide a more significant therapeutic effect, mainly by increasing lymphocyte subsets and immune responses, including T cell amplification, cytokine production and humoral response. These data support further evaluation of the combination of IL-7 and sip-T in larger clinical trials [114]. IL-12 is a well-known pro-inflammatory cytokine that activates NK and T cells to build a relationship between innate and adaptive immunity, thereby effectively increasing anti-tumor immunity [115]. The combination of IL-7 and IL-12 has better antitumor activity than cytokines alone. Nakao et al. designed a virus expressing cytokines IL-7 and IL-12 to stimulate the anti-tumor immune response. Via injection of the virus into tumor-bearing mice, the immune state of the former inflammatory immunogenic tumor was activated and led to complete tumor regression. Mice with complete tumor regression could resist rechallenge by the same tumor cell, suggesting that injection of the virus established long-term tumor-specific memory [13]. Meanwhile, dual expression of IL-7 and IL-12 increased the activation of CD8+T cells in poorly immunogenic tumors, contributing to better antitumor activity than IL-12 expression alone. In addition, the combination of IL-7 and IL-12 could synergistically stimulate T cells in tumors, upregulate various immune systems, enhance inflammatory states, and enhance anti-tumor effects. In addition, the combination of these two cytokines did not increase the number of percentage of exhausted CD8^+^ T cells, while IL-7 played a more important role in maintaining T cells in the tumor microenvironment [116]. The strategies mentioned above are shown in Figure 3B.

### 4.2. IL-7/IL-7R-Expressing CAR- or TCR- Modified T Cells Therapy

#### 4.2.1. IL-7-Expressing CAR-T and TCR T Cell in Cancer Immunotherapy

Although adoptive transfer of genetically engineered T cells expressing CAR or TCR has been extensively studied and developed for clinical applications, these methods need to be further improved, especially in terms of their efficacy. IL-7 is a vital cytokine that affects the survival of tumor-infiltrating T cells [117]. Currently, IL-7 has several applications in CAR-T research. A major cause of CAR-T cell failure is their limited expansion and persistence. A clinically compatible strategy was created to increase the production of B cell maturation antigen in CAR-T cells. The addition of IL-7/IL15 could increase CAR-T cells in the CD4+ Tscm portion and 70% of Tscm cells amongst CD8+ cells. This approach enabled CAR-T cells to achieve robust cell proliferation capacity for clinical use while maintaining effector function [118].

In addition, researchers have engineered CAR-T cells to secrete IL-7 and CCL19 (7 × 19). In vivo studies have shown that 7 × 19-secreting CAR-T cells exhibited higher antitumor ability than traditional CAR-T cells, eliminated the solid tumor, and increased the survival of mice [119]. Furthermore, 7 × 19 CAR-T cells showed increased expansion and migration in vitro, and greater antitumor activity in some cancer cell line and tissue samples. A phase 1 clinical trial was conducted in patients. In a patient with advanced hepatocellular carcinoma, the tumor disappeared completely after 30 days of injecting anti-glypican-3-7 × 19 CAR-T cells at the tumor site. In another patient with advanced pancreatic cancer, the tumor almost disappeared after 240 days of intravenous infusion with anti-mesothelin-7 × 19 CAR-T cells [120]. Triple negative breast cancer (TNBC) is one of the subtypes with poor prognosis due to the lack of specific targets. A recent study has shown that immunotherapy can overcome this problem by targeting folate acid receptor-α. Gene-modified γδT cells express the FRα-CAR and secrete IL-7 and CCL19 (7 × 19 CAR-γδT). The 7 × 19 CAR-γδT cells show anti-tumor activity in vitro, and are more beneficial to the growth of TNBC xenograft mouse models than CAR-γδT. This provides a possible therapeutic strategy for patients with refractory metastatic TNBC [121]. Based on its promising therapeutic effects, IL-7 expressing CAR-T cells are being used in various clinical trials for cancer immunotherapy (Table 1).

In addition to CAR-T, IL-7 also has anti-tumor effects in TCR T cells. Simultaneous expression of IL-7 and CCL19 in TCR T (7 × 19 P1A T) cells by genetic engineering can enhance the anti-tumor activity of TCR T cells by eliminating tumors and promoting long-term survival of mice. In addition, 7 × 19 P1A T cells produce potent and long-lasting anti-tumor activity in conjunction with PD-1 blockade therapy [122]. The strategies mentioned above for the use of IL-7 in cancer therapy are shown in Figure 3C.

#### 4.2.2. CAR-T Expressing Engineered IL-7R in Cancer Immunotherapy

IL-7R can be bound to IL-7 to activate downstream signaling pathways. Insertion of cysteine or proline leads to the homologous dimerization of IL-7R to form compositionally active IL-7R that can transmit IL-7 signals without ligand or common receptor γ chain. Once the homologous dimer is formed, the cross-phosphorylation of JAK1/JAK1 activates the downstream core signal node STAT5 of IL-7 [10,123]. Based on this feature, researchers constructed a constitutive signal mutant IL-7Rα by inserting cysteine into the transmembrane domain, and the extracellular domain of CD34 replaced the natural extracellular domain of the receptor. After modification, highly expressed and functionally active CD34-IL-7R was obtained and named C7R. C7R did not promote antigen-independent amplification of T cells in vitro. In other words, failure to maintain autonomic T cell amplification is an important feature of therapeutic safety. CAR-T cells co-expressing C7R were also active against metastatic neuroblastoma and glioblastoma [124]. In addition, C7R has been studied in TNBC. After co-expression of ALX-targeting CAR-T cells with C7R, its anti-tumor activity against TNBC cells was notably higher than that of traditional CAR-T cells and improved therapeutic efficacy and reduced tumor recurrence in TNBC xenograft model [125]. CAR-T cell treatment in patients with advanced solid tumors remains a major challenge. In a mouse model of human solid tumors, B7H3 CAR-T with PD-1 and C7R showed longer-lasting anti-tumor activity after fusion of the extracellular domain of the PD-1 receptor with the intracellular domain of C7R and binding to B7H3 CAR-T cells [126]. In another study, Anti-CD19-scFv CAR and TGF-β/IL-7R chimeric switch receptors were co-expressed in T cells. In vitro, it showed lower levels of SAMD2 phosphorylation and stronger target-specific cytotoxicity than the control CAR-T cells. In tumor-tolerant mouse models, overall survival and relapse-free survival were significantly longer in mice treated with CD19 CAR-Ttri-I7R-T cells than in control mice, providing a novel strategy for B cell lymphoma treatment [127]. All the above studies have shown that CAR-T cells expressing IL-7R can effectively kill specific cancer cells, crossing boundaries of traditional CAR-T cells in solid tumors, and provide a new strategy for cancer treatment. The strategies mentioned above are shown in Figure 3D. Based on its promising therapeutic effects, C7R-CAR-T cells are being used in various clinical trials for cancer immunotherapy (Table 1).

Furthermore, CTL that does not express IL-7Rα can be modified by gene editing to restore the response to IL-7, promote the cell proliferation, and have anti-tumor activity without significantly changing their antigen dependence and specificity [128]. The strategies mentioned above for the use of IL-7R pathway in cancer therapy are shown in Figure 3E.

### 4.3. Antagonist of IL-7R Pathway in Cancer Immunotherapy

In Section 3.2, the tumor-promoting effects of IL-7 and IL-7Rα and how they contribute to carcinogenesis have been described in detail. However, in the following section, we will detail the therapeutic use of IL-7R pathway antagonists in order to better understand their application in cancer immunotherapy.

#### 4.3.1. Anti-IL-7R Monoclonal Antibodies (Mabs) in Cancer Immunotherapy

Cancer is closely related to autoimmune diseases, and numerous curative antibodies are extensively used for the clinical treatment of these two diseases. IL-7R signal transduction play a crucial role in the advancement and progression of lymphoid malignancy and autoimmune diseases [129]. Anti-IL-7R neutralizing monoclonal antibodies have demonstrated therapeutic effect in preclinical research of autoimmune diseases, such as autoimmune diabetes [130] and arthritis [131]. The anti-IL-7R antibody-drug conjugate (A7R-ADC) has an anti-tumor effect on lymphatic malignancies and can target steroid-sensitive and -resistant cells; therefore, A7R-ADC could be a hopeful new choice for cancer treatment [132]. Common mutations in the IL-7R pathway may lead to T-ALL. A fully human anti-IL7Rα monoclonal antibody (B12) could recognize the wild-type and different gain-of-function mutant-driven T-ALL. B12 can inhibit IL-7R-mediated signaling pathway, promoting T-ALL cell death in vitro and delay the progression of leukemia in vivo, which has obvious preclinical value [133]. Two other anti-IL-7Rα monoclonal antibodies (4A10 and 2B8) that identify non-overlapping IL-7Rα epitopes, and can mediate antibody-dependent cell-mediated cytotoxicity and other repressive mechanisms and have curative effects on patient-derived xenograft T-ALL cells [134]. The strategies mentioned above for the use of IL-7R pathway in cancer therapy are shown in Figure 3F.

#### 4.3.2. IL-7R Pathway Signaling Inhibitors in Cancer Immunotherapy

The IL-7R pathway is a major driver of ALL, and in vitro studies have shown that the JAK1/2 inhibitor ruxolitinib inhibits ligand-independent signaling and induces death in transformed cell lines. In a mouse model of invasive leukemia developed by transformed cell lines, untreated mice died within three weeks, whereas adding ruxolitinib to the mouse diet effectively reduced leukemia symptoms and prolonged survival [135]. A phase 2 study of ruxolitinib combined with chemotherapy in childhood acute lymphoblastic leukemia was conducted to treat patients with an altered JAK-STAT pathway (NCT02723994). Ruxolitinib has also been used in combination with first-line treatment for acute lymphoblastic leukemia and lymphoma (NCT03117751). JAK inhibitors have also been used in combination with mTOR inhibitor and FLT3 inhibitors to treat ALL and AML [136,137]. Bcl-2 is an anti-apoptotic mediator downstream of the IL-7R signaling pathway that regulates cell survival. Venetoclax, an inhibitor of Bcl-2, is currently undergoing clinical trials for the treatment of hematological malignancies other than CLL. Venetoclax has shown anti-leukemia activity in vitro and strongly enhances ruxolitinib activity; however, the combination of the two drugs is more effective than either drug alone. Venetoclax is currently being used in preclinical trials to treat ALL (NCT03236857, NCT03181126). In addition, other models showed that ruxotinib combined with BCL-2 inhibitors had good therapeutic effects and future application prospects [138,139,140]. Because of this persistent phenomenon, new JAK inhibitors are being developed to reduce persistence. Everolimus is an mTOR inhibitor currently in trials to treat T-ALL ((NCT03328104, NCT03740334). The PI3K-MEK pathway is also associated with il-7R driving T-ALL [66,90,141]. In addition, mutations in IL-7R often occur in conjunction with Ras signaling to drive T-ALL [142]. Selumetinib is a potent and selective MEK1/2 allosteric inhibitor. Currently, an international trial of Selumetinib in combination with dexamethasone in the treatment of Ras mutation-driven T-ALL is underway, and more valuable clinical data will be available soon (ISRCTN92323261). The strategies mentioned above for the use of IL-7R pathway in cancer therapy are shown in Figure 3G.

## 5. Conclusions

Numerous studies have elucidated the basic physiological functions of IL-7 and IL-7Rα, including lymphocyte and lymph node development and peripheral T cell homeostasis. IL-7 facilitates immune reconstitution through thymopoiesis and thymus-independent homeostasis of peripheral T cells. IL-7/IL-7R have dual roles in the development of cancer, and exploring the IL-7R-mediated signaling pathways has shown good therapeutic effects in both liquid tumors and some solid tumor models, indicating a promising future for inhibiting tumor occurrence. These studies have paved the way for current and future translational and clinical studies and applications. However, the role of IL-7R-mediated signaling pathways in some solid tumors has yet to be confirmed. Extensive experiments are needed to verify the feasibility of IL-7 in cancer immunotherapy, as well as its side effects or other adverse effects.

## Figures and Tables

**Table 1:** Examples of ongoing clinical trials with IL-7 and IL-7R in cancer immunotherapy.

Drug	Interventions	Conditions	Status	Phases	NCT No.
IL-7	IL-7	Bladder Acute Myeloid Leukemia, Myeloproliferative Neoplasm	Recruiting	I	NCT04054752
IL-7	Atezolizumab + Glycosylated Recombinant hIL-7	Bladder Urothelial Carcinoma	Recruiting	II	NCT03513952
NT-I7	NT-I7	Recurrent Squamous Cell Carcinoma of Head and Neck	Recruiting	I	NCT04588038
NT-I7	NT-I7	AIDS-Related Kaposi Sarcoma	Recruiting	I	NCT04893018
NT-I7	NT-I7 + Pembrolizumab	Any Advanced Solid Tumors	Recruiting	I/II	NCT04332653
NT-I7	NT-I7 + Pembrolizumab	Recurrent glioblastoma	Not yet recruiting	II	NCT05465954
NT-I7	NT-I7 + atezolizumab	Non-Small-Cell Lung	Recruiting	II	NCT04984811
NT-I7	NT-I7 + Nivolumab	Gastric or Gastro-esophageal Junction or Esophageal Adenocarcinoma	Recruiting	II	NCT04594811
NT-I7	NT-I7 + Placebo	Malignant Glioma	Active, not recruiting	I	NCT02659800
NT-I7	NT-I7 + Placebo + Temozolomide + Radiation therapy	Newly diagnosed GBM	Recruiting	I/II	NCT03687957
NT-I7	NT-I7 + Kymriah^®^	Relapsed/Refractory Large B-cell Lymphoma	Recruiting	I	NCT05075603
NT-I7	NT-I7 + atezolizumab	High-Risk Skin Cancers	Recruiting	I/II	NCT03901573
IL-7 expressing CAR-T cells	CAR-T cell (expressing IL7 and CCL19)	Nectin4-positive Advanced Malignant Solid Tumor	Recruiting	I	NCT03932565
IL-7 expressing CAR-T cells	GPC3 and/or TGFβ targeting CAR-T cells (secret IL7/CCL19)	Hepatocellular Carcinoma with GPC3 expression	Recruiting	I	NCT03198546
IL-7 expressing CAR-T cells	CD19-7 × 19 CAR-T plus PD1 monoclonal antibody	Diffuse Large B-cell Lymphoma	Recruiting	I	NCT04381741
C7R-CAR-T	(C7R)-GD2.CART cells+ Cyclophosphamide+ Fludarabine	Diffuse Intrinsic Pontine Glioma High Grade Glioma Embryonal Tumor Ependymal Tumor	Recruiting	I	NCT04099797
C7R-CAR-T	(C7R)-GD2.CART cells+ Cyclophosphamide+ Fludarabine	Neuroblastoma, sarcoma, uveal melanoma, breast cancer, or another cancer that expresses a substance	Recruiting	I	NCT03635632
